# An Automated Instance Segmentation Method for Crack Detection Integrated with CrackMover Data Augmentation

**DOI:** 10.3390/s24020446

**Published:** 2024-01-11

**Authors:** Mian Zhao, Xiangyang Xu, Xiaohua Bao, Xiangsheng Chen, Hao Yang

**Affiliations:** 1 School of Rail Transportation, Soochow University, Suzhou 215006, China; 20215246020@stu.suda.edu.cn; 2 College of Civil and Transportation Engineering, Shenzhen University, Shenzhen 518061, China; bxh@szu.edu.cn (X.B.);; 3 School of Transportation and Civil Engineering, Nantong University, Nantong 226019, China

**Keywords:** crack detection, instance segmentation, deep learning, CrackMover

## Abstract

Crack detection plays a critical role in ensuring road safety and maintenance. Traditional, manual, and semi-automatic detection methods have proven inefficient. Nowadays, the emergence of deep learning techniques has opened up new possibilities for automatic crack detection. However, there are few methods with both localization and segmentation abilities, and most perform poorly. The consistent nature of pavement over a small mileage range gives us the opportunity to make improvements. A novel data-augmentation strategy called CrackMover, specifically tailored for crack detection methods, is proposed. Experiments demonstrate the effectiveness of CrackMover for various methods. Moreover, this paper presents a new instance segmentation method for crack detection. It adopts a redesigned backbone network and incorporates a cascade structure for the region-based convolutional network (R-CNN) part. The experimental evaluation showcases significant performance improvements achieved by these approaches in crack detection. The proposed method achieves an average precision of 33.3%, surpassing Mask R-CNN with a Residual Network 50 backbone by 8.6%, proving its effectiveness in detecting crack distress.

## 1. Introduction

Cracks are a common road distress that can lead to more severe road issues if not repaired promptly, such as compromising traffic safety and the intensified formation of potholes. These factors impact highway traffic services significantly, underscoring the importance of crack detection as a critical task in ensuring public safety and maintaining road quality. Traditional manual inspection methods require pavement maintenance workers to physically visit a site for inspection and measurements. However, this approach is labor-intensive, financially inefficient, and even poses risks to the workers. Therefore, the development of and research on automatic crack detection equipment hold significant value [1].

Numerous researchers have devoted their efforts to studying semi-automated methods for crack detection in recent decades [2]. Traditional approaches in digital image processing for crack recognition have relied primarily on techniques such as wavelet transform, image thresholding, manual feature and classification, and edge-detection-based methods. However, these digital image processing methods exhibit several drawbacks and limitations. Subirats et al., for instance, employed continuous wavelet transformations for crack detection [3], while Zhou et al. investigated a method for detecting, classifying, and evaluating hazardous road sections using wavelet transformation [4]. These wavelet-transform-based methods struggled to handle cracks with low continuity and high curvature. Thresholding is another popular method utilized for crack detection [5]. However, it is susceptible to various factors, such as road shadows, lighting conditions, road markings, and significant variations in crack depths. Although some researchers have attempted to improve the robustness of methods against noise [6,7], their generalizability and applicability still remain limited. Furthermore, traditional digital image processing techniques, including manual feature and edge detection [8,9], have gradually been replaced by newer methods due to the former’s heavy reliance on manual labor and vulnerability to noise.

The advancement in computer vision technology, particularly the continuous breakthroughs in the field of deep learning, has opened up new possibilities for automatic crack detection. Convolutional neural networks (CNNs) have gained significant popularity and proven to be highly efficient [10]. They initially excelled in image classification and gradually developed tasks such as crack detection, segmentation, and instance segmentation. They have also shown great potential in the detection of pavement defects [11,12,13]. By providing high-quality labeled images as input to the network, CNNs can automatically learn features with superior accuracy and generalization compared to traditional methods. Additionally, CNNs exhibit robustness against various disturbances, including uneven lighting, cluttered backgrounds, diverse crack shapes, and crack-like artifacts.

The focus in the initial stages of deep learning application in crack detection was on classification [14,15]. In this approach, pavement images were partitioned into image patches and provided as input to a CNN. Although this method yielded coarse outcomes and did not allow for the precise localization of cracks or characterization of fracture properties, it showcased the potential of deep learning in crack detection and stimulated further research in this area.

In recent years, CNN-based object detection methods for pavement distress detection have gained significant popularity. These methods can identify multiple categories of damage and provide approximate crack locations [16,17]. Liu et al. proposed a two-step crack detection and segmentation model which utilizes the You Only Look Once 3rd version (YOLOv3) for automatic crack detection, which exhibits high accuracy and serves as a foundation for subsequent work [18]. Mandal et al. introduced a YOLOv2-based pavement distress detection system capable of automatically detecting and classifying different types of cracks [19]. Xue et al. developed a tunnel lining defect detection and classification model based on a fully convolutional network (FCN) with an optimal accuracy exceeding 95%, more than the efficiency of Faster R-CNN and traditional methods [20]. Maeda et al. compiled a large pavement distress dataset for training and employed object detection methods to accurately classify distress into eight categories [21]. Considering that traditional detection methods are susceptible to the instability of real environmental backgrounds, Cha et al. presented a concrete crack identification technique based on CNNs. This method demonstrates superior performance compared to traditional edge detection techniques while being less affected by background noise [22]. However, object detection methods can only infer an approximate crack location and lack detailed features, thereby highlighting the necessity of pixel-level semantic segmentation.

The use of semantic segmentation for pavement distress detection has gained significant popularity over the past five years. Pixel-level prediction enables the accurate localization of cracks, quantification of crack widths, and extraction of various crack characteristics for further research [23,24,25]. Dorafshan et al. conducted a comparative study between traditional edge detection methods and deep CNN (DCNN)-based approaches for concrete crack detection. The results demonstrated that DCNN approaches achieved higher accuracy, finer precision, shorter computation time, and stronger noise resistance [26]. Alipour et al. proposed a pixel-level crack segmentation model called CrackPix, which utilizes deep, fully CNNs to provide pixel-level crack recognition by employing patch-level image classification that can operate on images of any size. This method outperformed traditional patch detection, edge detection, and thresholding approaches [27]. Dung et al. developed a concrete crack semantic segmentation model based on a deep FCN with a VGG16 backbone. This model accurately detects cracks and evaluates crack density [28]. Liu et al. introduced the use of U-shaped networks for the semantic segmentation of concrete cracks. This method exhibits robustness and accuracy under challenging conditions, such as uneven lighting, inconsistent backgrounds, and varying crack widths [29]. Ren et al. proposed a pixel-level segmentation model called CrackSegNet for concrete cracks. It is an enhanced deep, fully CNN that offers higher accuracy and better generalization compared to traditional image processing techniques [30]. Semantic segmentation allows for the extraction of detailed crack features, including direction, position, length, and width.

**Figure 1:**
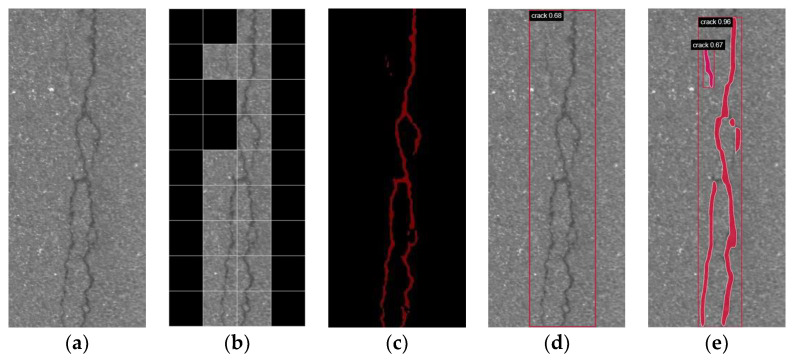
Comparison of various computer vision tasks: (**a**) crack image; (**b**) classification; (**c**) semantic segmentation; (**d**) object localization; (**e**) instance segmentation.

However, the direct application of intensive semantic segmentation on high-resolution pavement images can lead to a notable decrease in accuracy. To mitigate this issue, a potential strategy is to incorporate localization and segmentation, commonly referred to as instance segmentation. Figure 1 provides a comprehensive visual representation of the instance segmentation approach compared to existing methods. The original image is depicted in Figure 1a. Subsequently, Figure 1b illustrates crack classification, wherein the image is partitioned into smaller patches, and the presence of cracks in each patch is determined. Figure 1c demonstrates semantic segmentation, which involves segmenting cracks and backgrounds, thereby enabling the pixel-level prediction of cracks within the image. Moreover, Figure 1d showcases crack localization, wherein bounding boxes encompassing the cracks are overlaid, accompanied by corresponding labels. Finally, Figure 1e exemplifies instance segmentation, which not only predicts the category and assigns the appropriate label to each instance but also employs pixel-level masks to precisely delineate the position of each instance. Currently, only a limited number of studies have delved into pavement crack instance segmentation [13,31,32], yet significant challenges persist, primarily stemming from the deficiency of pavement instance segmentation datasets and the shortcoming of large-size detection capability.

The actual detected pavement image may encompass a significantly larger area compared to the depiction shown in Figure 1. To ensure the attainment of effective detection results, this paper contributes in the following ways:

A data augmentation scheme specifically designed for crack detection is proposed which leverages the limited variability within a small mileage and the continuity of pavement images, called CrackMover. It expands the dataset using existing images and their corresponding manual annotations by cropping and randomly transforming them. The experimental results show that our approach achieves a maximum improvement of 4.1% in average precision (AP). 

A novel instance segmentation method customized for crack detection is presented, which combines localization and segmentation functions. It employs the ConvNeXt [33] backbone and leverages the cascade structure [34] for the R-CNN part.

By combining these techniques, our proposed method achieves state-of-the-art outcomes, outperforming Mask R-CNN by 8.6% in terms of AP.

## 2. Methodology

Pavement images are typically captured using continuous shooting and then cropped into individual images. Notably, for a small range of pavement (e.g., within a single image or adjacent images), the differences are typically negligible. Therefore, a novel data augmentation method is developed to construct pseudo-images by cropping and transforming a crack instance on the image and inpainting it with pixels in adjacent locations, called CrackMover. Specifically, it builds new crack instances by clipping crack annotations, then panning and stochastic flipping them to generate new training samples. In this section, an overall framework for crack instance segmentation is also introduced, which leverages the ConvNeXt backbone network and cascade technology to improve the accuracy of crack detection. 

### 2.1. CrackMover

**Figure 2:**
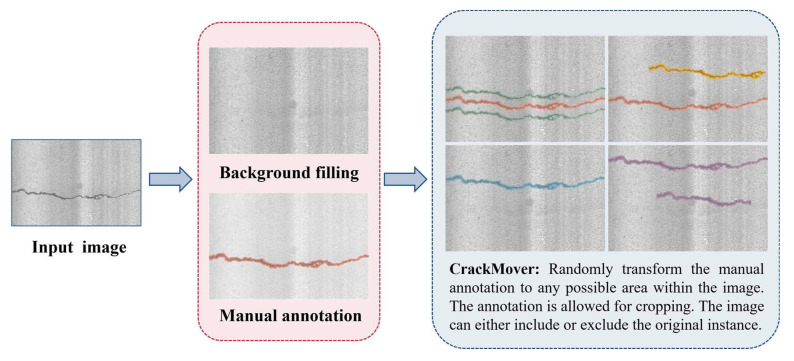
Detailed description of CrackMover. The rose–red color annotation in the image represents the original instance, and the other colors represent the added instances.

CrackMover randomly selects images from a dataset and applies random transformations to their existing mask annotations, thus generating new images and expanding the dataset. A detailed introduction of CrackMover is shown in Figure 2. CrackMover consists of two main steps: (1) Instance preparation and background filling, where cracks and backgrounds are separated to prepare for subsequent transformations. There are two ways to augment the data: either by adding the target instance at an appropriate location while retaining the original target instance, or by removing the original target instance and adding it to another appropriate location. The latter method requires background fill, which is achieved by filling the gap with pixels from adjacent spaces, given that the pavement images in the dataset are taken continuously and the background is relatively uniform. (2) The random transformation of target instances, where the target instance patch is extracted from a selected sample in the dataset, and its position on the sample image is defined by the transform matrix of Equation (1):

(1) $$H=\left[\begin{array}{ccc} \sigma & 0 & {∆}_{x}\\ 0 & \sigma & {∆}_{y}\\ 0 & 0 & 1 \end{array}\right]$$

where ${∆}_{x}$ and ${∆}_{y}$ are real numbers representing the amount of displacement on the x and y axes, and σ is a real number representing the scale variance.

CrackMover can be seamlessly integrated into a PyTorch training pipeline and provides an efficient and effective way to increase the variability of the input data during training, thereby improving the robustness and generalization of the trained model.

### 2.2. Overall Framework

**Figure 3:**
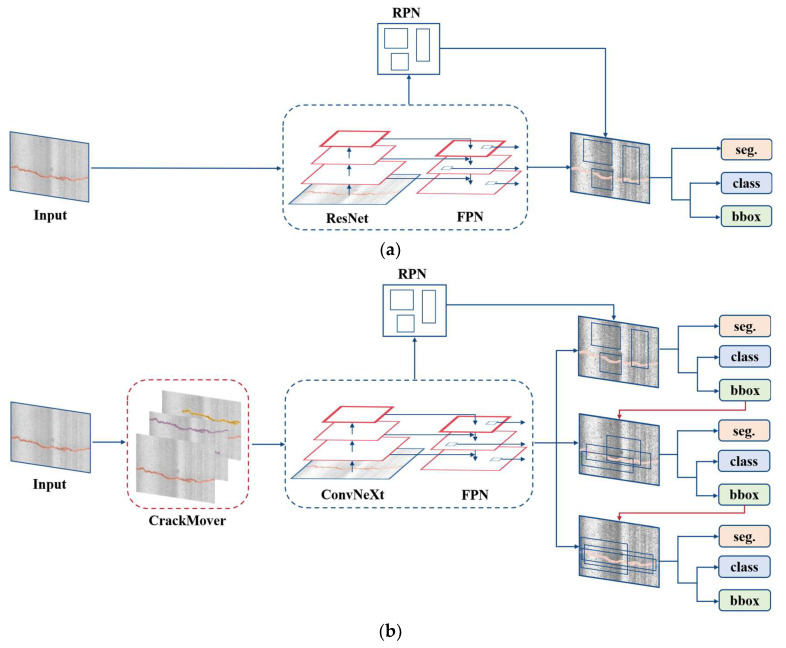
Training pipeline of Mask R-CNN and our proposed method. Seg. is segmentation. (**a**) Mask R-CNN. (**b**) Proposed method.

Figure 3 shows a comparison of the proposed method with the most commonly used Mask R-CNN. During the training phase, crack images first perform data augmentation through CrackMover, a plug-and-play module that is not included in the commonly used Mask R-CNN. Then, they are fed into the backbone network for feature extraction, which in this case is ConvNeXt, not ResNet. The resulting feature maps are then fused using Feature Pyramid Networks (FPNs) [35]. These fused feature maps are subsequently input into five identical Region Proposal Networks (RPNs), which perform classification and bounding box (bbox) regression calculations. The RPNs generate region proposals, which define the regions of interest (RoIs). The RoI feature maps are then passed through RoIAlign for consistent and accurate sampling. Different from Mask R-CNN, a cascade structure is employed to enhance the detection performance. Each cascade detector performs secondary screening on the output of the previous detector and finally inputs it into the fully connected layers for classification and regression calculation. Notably, in the last stage of the cascade, a mask prediction head is added to enable mask prediction.

#### 2.2.1. Backbone

**Figure 4:**
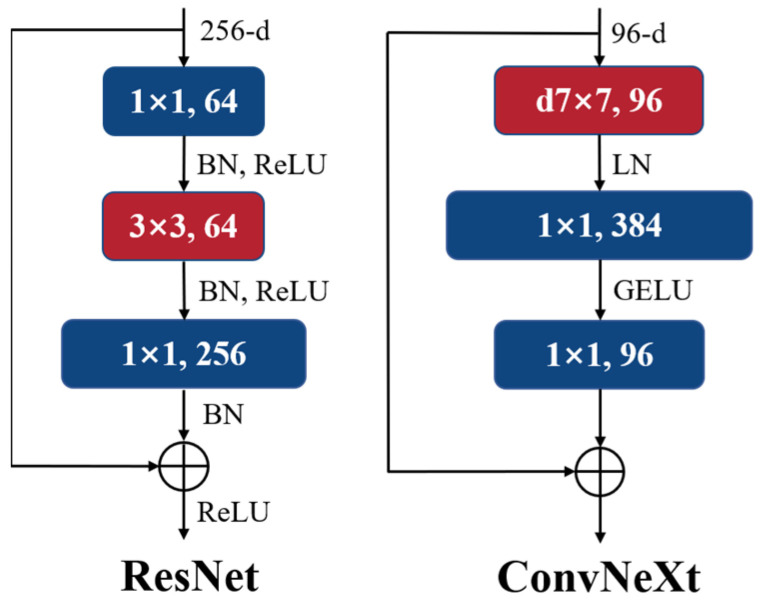
Comparison between ResNet and ConvNeXt.

The ConvNeXt architecture serves as the backbone of the network [33], which is an enhancement of the ResNet architecture [36]. The overall structure of the network remains unchanged, but the training strategy and design principles of the SwinTransformer network, which has gained popularity recently, are applied to training ResNet. By combining the strengths of both architectures, the performance of the traditional convolutional neural networks is improved. It is worth mentioning that this method makes ResNet perform better than SwinTransformer under the same training strategy and design principles. Five key strategies are employed, which are detailed below. A comparison of the block structures between ResNet and ConvNeXt is illustrated in Figure 4.

Macro-Design: The stage compute ratio of SwinTransformer tiny (Swin-T) is set as 1:1:3:1. To align with this ratio, the number of blocks in each stage of ResNet is adjusted accordingly from (3, 4, 6, 3) to (3, 3, 9, 3). The stem cell of SwinTransformer utilizes a “patchify” strategy with a patch size of four. Similarly, in ConvNeXt, the stem cell of ResNet is substituted with a “patchify” layer, where the original 7 × 7 convolutional layer with a stride of two is substituted with a 4 × 4 convolutional layer with a stride of four.

ResNeXt: It incorporates the core idea of “group convolution, expand width” from ResNeXt, which enables the network to better balance floating point operations per second (FLOPs) and accuracy. In this case, depthwise convolution with the same number of groups as channels is utilized, effectively reducing FLOPs while slightly sacrificing accuracy. Furthermore, the network width is increased from 64 to 96, enhancing network accuracy but unavoidably increasing FLOPs.

Inverted Bottleneck: This part is the same as SwinTransformer, referring to MobileNetV2, with an inverted bottleneck structure. This means the hidden dimension of the Multi-Layer Perceptron (MLP) is four times larger than the input dimension. Although the last point increases FLOPs, the network’s FLOPs are greatly decreased by the design of this point.

Large Kernel Size: A large convolutional kernel will be used. Depthwise convolution is moved up in the network, placing the more complex and inefficient multi-head self-attention (MSA) block ahead of the efficient and dense MLP block. This arrangement aligns with the inverted bottleneck structure mentioned earlier. The kernel size is increased from 3 × 3 to 7 × 7, resulting in optimal performance for the network.

Micro-Design: The Rectified Linear Unit (ReLU) is replaced with the smoother Gaussian Error Linear Unit (GELU) activation function. Following the Transformer architecture, only one GELU activation function is used in each block, reducing the number of activation functions compared to ResNet. Similarly, the normalization layer is simplified, retaining only one BatchNorm (BN) layer after the depthwise convolution. Additional BN layers do not improve accuracy, and Layer Normalization (LN) is used as a replacement. A separate downsampling layer strategy is implemented, employing 2 × 2 convolutional layers with a stride of two for downsampling.

#### 2.2.2. Cascade Mask R-CNN

Cascade Mask R-CNN [34] is a widely recognized and high-performance instance segmentation algorithm. In our proposed method, the R-CNN structure from Cascade R-CNN is adopted. The main objective of the cascade structure is to address the limitation of the intersection over union (IoU) threshold selection in the R-CNN part. This limitation often leads to increased noise and negatively impacts the detection performance. Figure 3 illustrates the cascade structure. The R-CNN part of our method comprises three detectors with progressively increasing IoU thresholds: 0.5, 0.6, and 0.7. At each stage, the detector is re-sampled to ensure an equal number of positive samples. The output of the previous detector will be the input to the latter detector.

**Figure 5:**
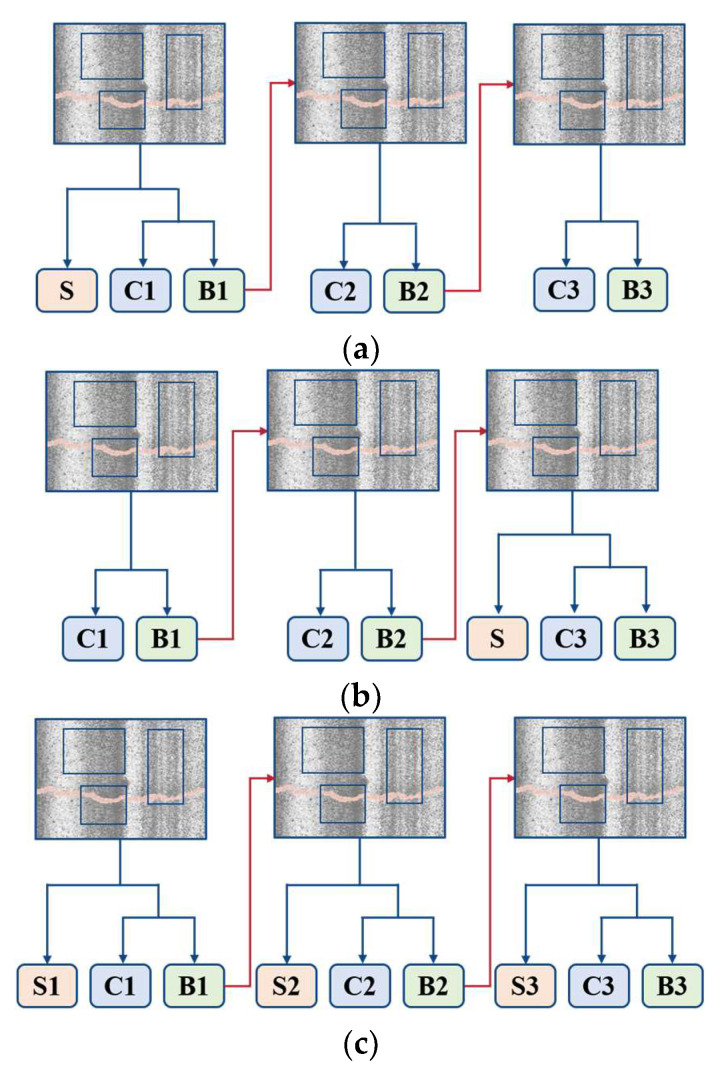
Three different Cascade Mask R-CNN strategies. S is segmentation, C is classification, and B is bounding box. (**a**) Segmentation in the first stage. (**b**) Segmentation in the last stage. (**c**) Segmentation in every stage.

The mask part of Cascade Mask R-CNN shares similarities with Mask R-CNN [37]. It introduces one parallel segmentation branch alongside the current detection branch in the network pipeline. There are three strategies for the location of segmentation branches, as shown in Figure 5, (a) to add a segmentation branch in the first stage of Cascade R-CNN, (b) to add a segmentation branch in the last stage of Cascade R-CNN, and (c) to add segmentation branches at each Cascade R-CNN stage. The experimental results of [34] prove that the effect of strategy (c) is the best. Therefore, the R-CNN structure of our proposed method is shown in Figure 5c.

The loss function of the network can be expressed as follows:

(2) $$L_{cls}=-\sum \left(Clog\left(P_{cls}\right)+(1-C)log\left(1-P_{cls}\right)\right)$$

Here, C represents the ground truth class labels, and $P_{cls}$ denotes the predicted class probabilities. The bounding box regression loss, denoted as $L_{reg}$, is calculated as follows:

(3) $$L_{reg}=\sum {smooth}_{L_{1}}\left(B-B_{gt}\right)$$

Here, B represents the anticipated bounding boxes, and $B_{gt}$ is the ground truth bounding boxes. The mask segmentation loss, denoted as $L_{mask}$, is calculated using the binary cross-entropy function, as follows:

(4) $$L_{mask}=-\sum \left(Mlog\left(P_{mask}\right)+(1-M)log\left(1-P_{mask}\right)\right)$$

Here, M represents the ground truth mask targets, and $P_{mask}$ represents the predicted masks. These loss components are combined using weight coefficients, such as $\;\lambda_{1}$ and $\lambda_{2}$, to form the total loss function, denoted as $L_{total}$:

(5) $$L_{total}=L_{cls\;}+\lambda_{1}L_{reg}+\lambda_{2}L_{mask}$$

## 3. Implementation Details

In this section, the details of the experiment will be introduced, such as the dataset, experimental platform, training strategy, hyperparameters, optimization strategy, etc.

### 3.1. Dataset 

**Figure 6:**
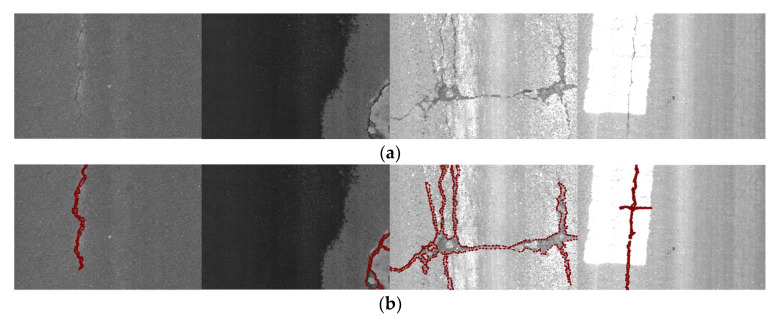
Crack images in the dataset and their annotations: (**a**) crack images in the dataset; (**b**) annotations of images.

The crack dataset for this paper was collected from actual roads using professional cameras, and the acquisition process was continuous. The crack images in the dataset are shown in Figure 6a, and their corresponding mask annotations are shown in Figure 6b.

The dataset used in this experiment was collected from real roads. The original dataset comprises 1844 images depicting various road surfaces. Through preprocessing work such as recutting and picking, the dataset has been augmented to a total of 2215 images, all showcasing pavement with cracks. Each image has dimensions of 1500 × 1000 pixels. To facilitate the training and evaluation of our model, the dataset is partitioned into training data and testing data. Approximately 90% of the dataset is assigned to the training data, and the remaining 10% is allocated to the testing data. Unless otherwise noted, all the models used for the experiments are pre-trained on ImageNet. To enable instance segmentation, the dataset is annotated with instance mask labels. These annotations are converted into the COCO format, a widely used format for, for instance, segmentation tasks. The cracks in the dataset encompass a range of crack types, including transverse cracks, longitudinal cracks, reticulated cracks, and more. Moreover, the images exhibit various forms of disturbance, such as ponding water, joints, low contrast, and road marking lines. By including these diverse and challenging scenarios, the aim is to enhance the robustness and generalization capabilities of our model.

### 3.2. Experiment Platform

For the implementation of our network, PyTorch version 1.8.0 is utilized, a widely adopted deep learning framework. The training process is performed on an NVIDIA RTX 3060 GPU, leveraging its computational capabilities. The operating platform used is Ubuntu 20.04, with CUDA version 11.1 for GPU acceleration. To optimize the training process, the hyperparameters are carefully selected. The learning rate is selected as 0.005, controlling the step size during parameter updates to find an optimal solution. A weight decay of 0.05 is applied to introduce regularization and mitigate overfitting. The training is conducted for 48 epochs, indicating the number of complete passes through the entire dataset. Each training iteration processes a batch size of 16 samples before changing the model parameters, which impacts the number of pictures conveyed through the network. The AdamW optimizer, batch normalization, warm-up, etc., are also used to improve the training results.

## 4. Experiments and Results

In this part, a comprehensive description of the experimental setup and results will be provided. The comparison study will be discussed, including the baselines used, the evaluation index results, the visualization results of both the baselines and our proposed method, the ablation learning experiments, and the P–R (Precision–Recall) curve analysis. By providing a detailed description of the experiments, the aim is to give a thorough and meaningful analysis of our method.

### 4.1. Baselines

Eight currently popular instance segmentation algorithms are reproduced and tested with the same crack dataset. The baselines are carefully selected to represent modern approaches in the area of instance segmentation, namely Mask R-CNN [37], Mask Scoring R-CNN [38], Cascade Mask R-CNN [34], PointRend [39], QueryInst [40], Hybrid Task Cascade [41], YOLACT [42], and SCNet [43]. The baselines are briefly described as follows.

Mask R-CNN and its variants: Mask R-CNN is a widely adopted architecture that extends the Faster R-CNN [44] architecture with an additional mask branch to generate pixel-level segmentation masks. Mask Scoring R-CNN extends Mask R-CNN through introducing one mask scoring mechanism to evaluate the quality of predicted masks. This allows for more reliable instance segmentation results by filtering out low-quality masks. Cascade Mask R-CNN was introduced in Section 2.2.2.

PointRend: It introduces a point-based sampling strategy for refining the mask predictions. By selectively processing a subset of pixels within each instance, it achieves more detailed and precise segmentation results.

QueryInst: It proposes a query-guided instance segmentation framework. It employs a query module to generate query-specific representations, facilitating accurate localization and segmentation of instances, even in crowded scenes.

Hybrid Task Cascade: It adds a task branch for semantic segmentation to Cascade R-CNN architecture. It effectively integrates instance-level and semantic-level information to improve instance segmentation performance.

YOLACT: It introduces a fully convolutional framework for real-time instance segmentation. It adopts a single-shot detection paradigm and employs a set of learnable masks to achieve fast and accurate instance segmentation results.

SCNet: It proposes a semantic-conditioned network that leverages semantic information for instance segmentation. It utilizes semantic context to guide the instance-aware representation learning process, leading to improved segmentation accuracy.

### 4.2. Comparison Study of Our Proposed Method against Baselines

**Figure 7:**
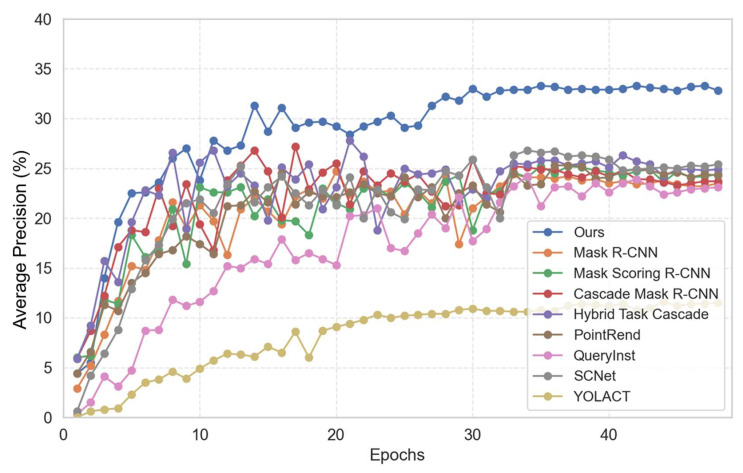
Changes in AP with epochs for our proposed method compared to baselines.

For the baselines in Table 1, the AP, AP50, and AP75 are calculated as evaluation indicators, where the AP is the average of every 0.05 AP with an IoU from 0.5 to 0.95, AP75 is the average precision specifically computed at an IoU threshold of 0.75, and AP50 corresponds to the average precision at an IoU threshold of 0.5. The AP results of our proposed method compared to the baselines are shown in Table 1, and the changes in the AP with epochs for our proposed method compared to those of the baselines are shown in Figure 7.

The findings reported in Table 1 show that the comprehensive effect of our proposed method outperformed that of the baselines. To ensure fairness in the experiment, ResNet-101 was chosen as the backbone for the baselines. Among the evaluated algorithms, Cascade Mask R-CNN demonstrates the best overall performance. The corresponding entry in Table 1 is highlighted in italics. The AP of Cascade Mask R-CNN is 27.2%, which is 2.5% greater than that of the classical algorithm Mask R-CNN. Although the results show that Hybrid Task Cascade is slightly better than Cascade Mask R-CNN, its computational effort is larger than that of Cascade Mask R-CNN. Considering that a cascade structure is selected as the basis for our proposed method Hybrid Task Cascade is improved based on Cascade Mask R-CNN, which can prove the superiority of the cascade structure in any case. The values highlighted in bold in the table represent the outcomes obtained by our proposed method, which outperforms the baselines. Specifically, our method achieves an AP of 33.3%, an AP50 of 59.9%, and an AP75 of 35.8%. These results surpass Cascade Mask R-CNN by 6.1%, 7%, and 10.3%, respectively. The AP changes with epochs for our proposed method compared to the baselines are shown in Figure 7, with each experiment trained for 48 epochs. Our proposed method significantly outperforms eight baselines and reaches a peak of 33.3% at epoch 35. The superior performance of our approach is evident from the evaluation metrics obtained through experimentation.

**Figure 8:**
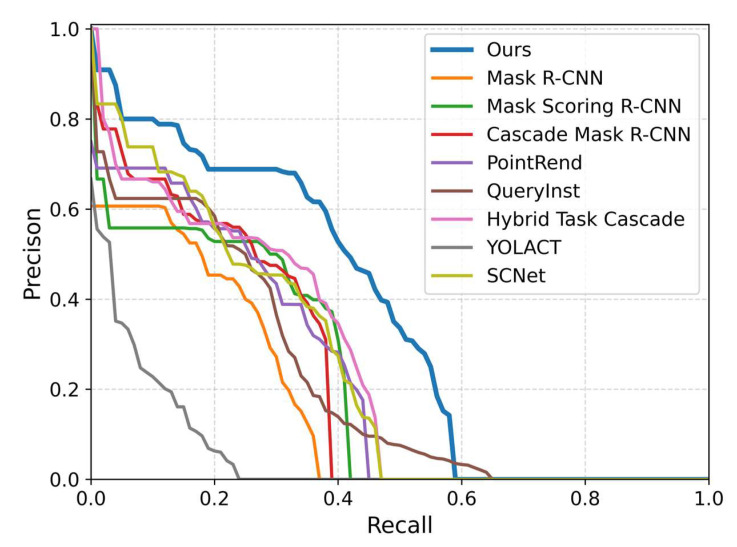
P–R curves of our proposed method compared to baselines.

The P–R curve is a widely used metric for evaluating detector performance, as it illustrates the relationship between recall and precision at various thresholds. A larger area under the P–R curve indicates better detector performance. In Figure 8, the P–R curve of our approach is compared with those of the baselines, including Mask R-CNN, Mask Scoring R-CNN, Cascade Mask R-CNN, PointRend, QueryInst, Hybrid Task Cascade, YOLACT, and SCNet. By analyzing the P–R curves, the performance of each method across different recall and precision thresholds can be assessed. Figure 8 compares the P–R curve of our approach to that of the baselines.

In Figure 8, the P–R curves of various approaches are compared, specifically at an IoU threshold of 0.75. The curves are color-coded for clarity: our proposed method is represented by the blue curve, Mask R-CNN by the orange curve, Mask Scoring R-CNN by the green curve, Cascade Mask R-CNN by the red curve, PointRend by the purple curve, QueryInst by the brown curve, Hybrid Task Cascade by the pink curve, YOLACT by the gray curve, and SCNet by the yellow curve. Upon careful examination, it is evident that our proposed method has the biggest enclosed area under its curve of any of the baselines, signifying superior performance.

### 4.3. Visualization Results of Comparison Study 

**Figure 9:**
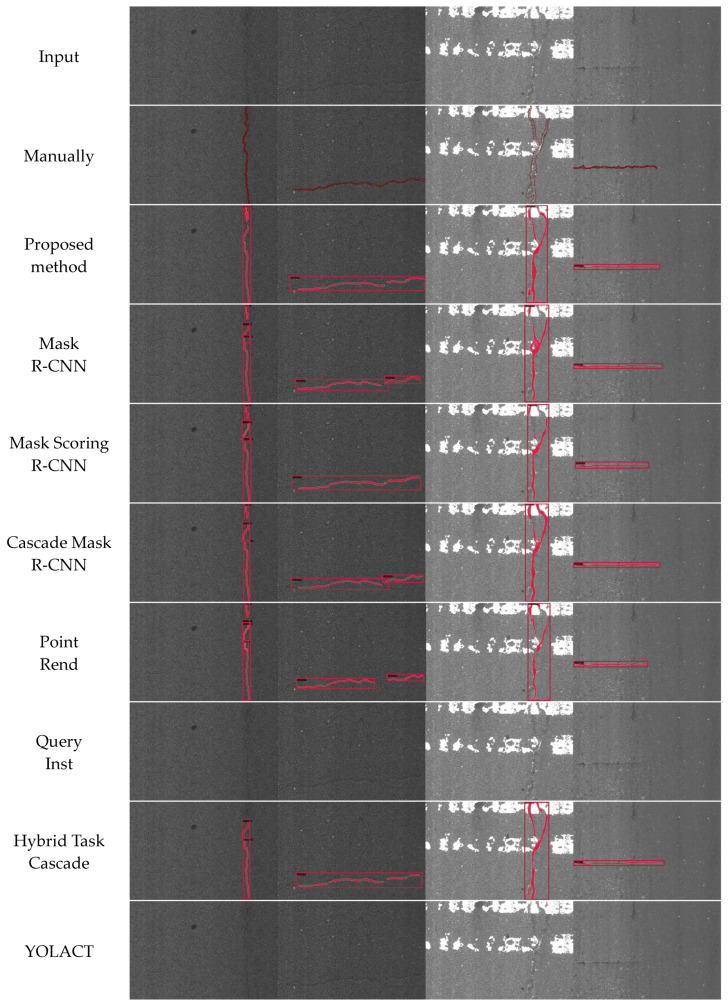

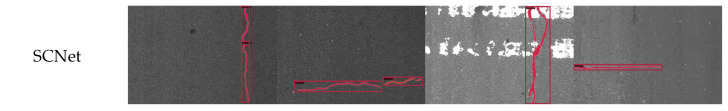
Crack instance segmentation results compared to baselines. From top to bottom: input, manual labels, our proposed method, Mask R-CNN, Mask Scoring R-CNN, Cascade Mask R-CNN, PointRend, QueryInst, Hybrid Task Cascade, and YOLACT, SCNet.

In this section, the visualization results of crack detection obtained by our proposed method and the baselines are presented, including Mask R-CNN, Mask Scoring R-CNN, Cascade Mask R-CNN, PointRend, QueryInst, Hybrid Task Cascade, YOLACT, and SCNet. For comparison purposes, four representative images are randomly selected from the testing dataset. Figure 9 shows the crack detection outputs generated by each method for the selected images. By visually inspecting the results, the performance and effectiveness of our proposed method in accurately identifying and delineating cracks can be compared with the baselines.

In Figure 9, the selected four representative images exhibit various characteristics, including horizontal cracks, longitudinal cracks, bifurcated cracks, complex backgrounds, cracks resembling the background threshold, the presence of zebra crossings, etc. The instance segmentation results of our proposed approach demonstrate high completeness, including accurate positioning of masks and bounding boxes. Among the baselines, Cascade Mask R-CNN achieves more comprehensive crack detection, and Hybrid Task Cascade is comparable to it. However, the accuracy of bounding box positioning falls short compared to our proposed method. Mask R-CNN misses the detection of bifurcated cracks and struggles to accurately locate bounding boxes for cracks resembling the background threshold. Mask Scoring R-CNN exhibits a few missed detections for bifurcated crack masks. PointRend and SCNet have less accurate bounding box positions. In the case of QueryInst and YOLACT, they demonstrate limited effectiveness as no cracks are detected in the four selected images. Comparing our suggested approach to the baselines demonstrates its great performance.

### 4.4. Ablation Study

**Figure 10:**
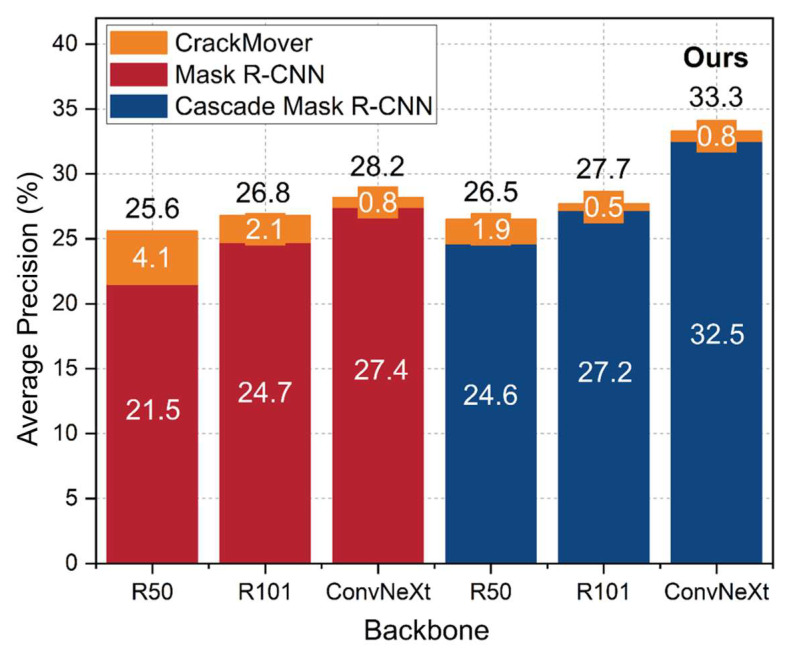
Comparing crack instance segmentation methods with different settings.

**Figure 11:**
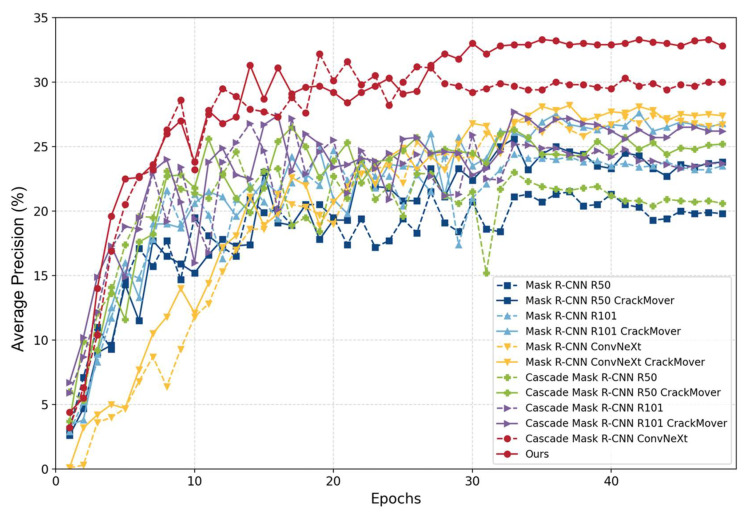
AP changes with epochs for crack instance segmentation methods: CrackMover vs. no CrackMover, cascade vs. no cascade, and different backbone networks.

In this part, the impact of three key design components in our proposed method is investigated: CrackMover, the ConvNeXt backbone, and the cascade structure. Following the same dataset used in the previous experiments, ablation experiments are conducted to evaluate the AP. The backbone network (ResNet-50, ResNet-101, and ConvNeXt) and the cascade structure are evaluated separately, along with the CrackMover module. To thoroughly analyze the effects of these design components, twelve network models are conducted and compared. Each model incorporates a new module, which is then retrained, and their AP scores are analyzed and compared. The results of these experiments are depicted in Figure 10, and the changes in the AP with epochs of these experiments are shown in Figure 11. It presents the APs obtained by the different network models, allowing us to assess the contributions of the CrackMover module, the ConvNeXt backbone, and the Cascade R-CNN structure individually. In addition, the changes in AP with epochs can be observed for each experiment.

According to Figure 10, the abscissa represents different backbone networks, namely ResNet-50, ResNet-101, and ConvNeXt, while the ordinate represents AP. In the figure, red is Mask R-CNN, blue is Cascade Mask R-CNN, and orange represents the increase in AP when the CrackMover module is added to the network while keeping other parts unchanged. The histogram results indicate that Cascade Mask R-CNN outperforms Mask R-CNN for the same backbone network. Specifically, for ResNet-50, ResNet-101, and ConvNeXt, Cascade Mask R-CNN achieves AP scores that are 3.1%, 2.5%, and 5.1% higher than Mask R-CNN, respectively, reaching 24.6%, 27.2%, and 32.5%. The cascade structure implemented in Cascade Mask R-CNN, where three identical detectors are cascaded to gradually increase the IoU threshold, proves to be effective in improving performance. Among the different backbone networks, both Mask R-CNN and Cascade Mask R-CNN perform best when using ConvNeXt, followed by ResNet-101 and ResNet-50. For Mask R-CNN, using ConvNeXt yields AP scores that are 2.7% higher than ResNet-101 and 5.9% higher than ResNet-50; for Cascade Mask R-CNN, using ConvNeXt results in AP scores that are 5.3% higher than ResNet-101 and 7.9% higher than ResNet-50. ConvNeXt is an improvement based on ResNet. The results show that training ResNet with the training strategy and design ideas of SwinTransformer yields significant improvements, even surpassing the performance of SwinTransformer itself. Finally, the CrackMover module, a data augmentation method for crack instance segmentation, demonstrates a clear positive effect on network performance. When the CrackMover module is added to each network with the other parts unchanged, the AP increases, with the highest increase reaching 4.1%. Our proposed approach scores 33.3% in terms of AP, 11.8% better than Mask R-CNN with ResNet-50.

To study the variation in the experimental AP with epochs, the results are described in a line graph, as shown in Figure 11. The dotted line in the figure indicates that the CrackMover module is not added, and the solid line indicates that the CrackMover module is added. dark blue represents Mask R-CNN with ResNet-50; light blue represents Mask R-CNN with ResNet-101; yellow represents Mask R-CNN with ConvNeXt; green represents Cascade Mask R-CNN with ResNet-50; purple represents Cascade Mask R-CNN with ResNet-101; and red represents Cascade Mask R-CNN with ConvNeXt. The results of this graph are the same as the above conclusions. Each experiment is trained for 48 epochs, and the backbone network performed ConvNeXt better than ResNet-101 and ResNet-50 throughout the training process; adding a cascade structure and CrackMover module is better than not adding them.

### 4.5. Visualization Results of Ablation Study

**Figure 12:**
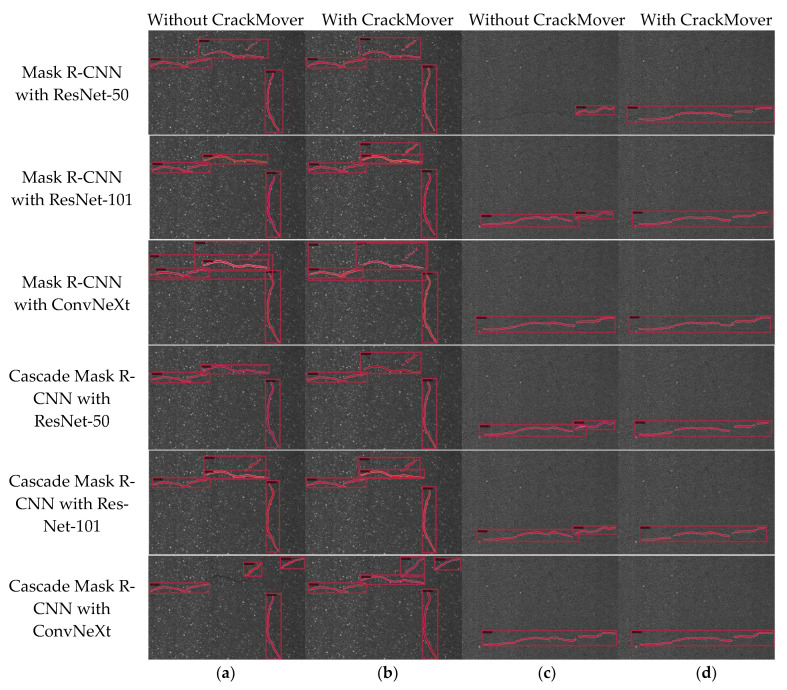
Crack instance segmentation results of different ablation settings. From top to bottom: Mask R-CNN with ResNet-50, Mask R-CNN with ResNet-101, Mask R-CNN with ConvNeXt, Cascade Mask R-CNN with ResNet-50, Cascade Mask R-CNN with ResNet-101, and Cascade Mask R-CNN with ConvNeXt. (**a**,**c**) Crack instance segmentation results without CrackMover, (**b**,**d**) crack instance segmentation results with CrackMover.

The visualization results of the ablation study are presented, investigating the role of three key components of our proposed method: CrackMover, the ConvNeXt backbone, and the cascade structure. To thoroughly analyze the effects of these design components, twelve network models are studied and compared. Each model contains a new module, the backbone network (ResNet-50, ResNet-101, or ConvNeXt), cascade structure, and CrackMover, which are evaluated separately. Two crack images are randomly selected in the testing data for evaluation. The visualization outcomes are shown in Figure 12.

In Figure 12, the visualization results of the ablation study are shown. Two representative images of cracks are selected in the testing data, which have the following characteristics: multi-cracks, bifurcated cracks, transverse cracks, longitudinal cracks, cracks and background threshold approximations, etc. In this set of images, the horizontal comparison shows that in the same row, that is, the same method and the same backbone network, the effect of (b) and (d) is better than that of (a) and (c), and the cracks that can be detected are more complete because (b) and (d) are the results after adding CrackMover. From the longitudinal comparison, we can observe that Cascade Mask R-CNN exhibits superior performance compared to Mask R-CNN; ConvNeXt is better than ResNet-50 and ResNet-101, with fewer missed detections; and the location of masks and bounding boxes is more accurate. In the end, it is evident from the instance segmentation visualization that the outcomes of our proposed method are the most complete, and the visual results show that the performance of our method is better than all the other models, and every module improved its effectiveness.

## 5. Discussion

It is an inevitable trend to implement instance-segmentation-level detection for cracks, as there are often other disturbances on the road, such as water stains, ruts, and other garbage. Traditional semantic segmentation methods, which segment only a single category, are often prone to misjudgment and can provide inaccurate information for maintenance purposes. Our work explores pavement distress detection at the instance segmentation level, but there is still a lot of room for development. One key challenge we faced in our work was the high cost of labeling instance segmentation data. To address this issue, we developed a data augmentation scheme customized specifically for pavement detection tasks. This allowed us to improve the detection performance of our method without relying on network architecture design, even when the same amount of labeled data was used. Secondly, high-precision cameras provide clearer images but also make the image resolution higher and the proportion of pixels occupied by cracks smaller. This makes our task more difficult to discern than the general visual domain, which often acts as an instance of subject status. Therefore, we chose the cascade structure to deal with the relatively small cracks. The results show that the use of the cascade structure is a very effective method for this task. We also found that cascade structures with stronger backbone networks could achieve better results (without a cascade structure: 2.7% average precision (AP) improvement from ResNet-101 to ConvNeXt; with a cascade structure: 5.3% AP improvement from ResNet-101 to ConvNeXt). This means that if a stronger backbone network emerges, a more pronounced effect can be achieved. Lots of results show that these traditional methods are ineffective in detecting the target of this special form of crack and cannot form an effective detection, and the anchor-based methods may still be the mainstream of crack detection methods for some time to come, as evidenced by recent studies [45].

One of the main contributions of this paper, CrackMover, considers the characteristics of pavement images and, thus, a very simple data augmentation method is designed. The way CrackMover operates enables it to adapt to most distress and background noise. When there are shadows or other noises on the pavement, moving and exchanging distress and a noisy background, it should still be able to construct new images. Unfortunately, the dataset in this paper does not contain many images with noisy backgrounds, but previous research has shown that deep learning has good generalizability [13,46]. In the future, more efficient and effective data augmentation techniques will be explored to address the limitations of crack images encountered in real-world scenarios, especially considering more a priori information about cracks. Additionally, a more suitable crack detection framework will be developed to handle complex situations, such as high background noise, interference from road markings, and cracks resembling the background threshold. Furthermore, the detection results of masks and bounding boxes will enable the calculation of detailed crack characteristics, including width, length, density, and other relevant parameters.

## 6. Conclusions

In this study, we introduced a novel deep-learning-based approach for crack instance segmentation with the aim of facilitating road maintenance management and improving operational efficiency. Our proposed method not only accurately localizes cracks but also achieves pixel-level segmentation. The experimental evaluations have demonstrated that our method surpasses state-of-the-art performance in real-world pavement detection scenarios. The primary innovation of our approach lies in the introduction of the CrackMover data augmentation technique, which leverages the inherent structural similarity characteristics of a pavement within a limited range. This method effectively augments the dataset through a straightforward approach and incurs only a minimal increase in computational costs while not adding to the inference overhead. By combining this technique with the ConvNeXt backbone network and a cascade structure, our approach achieves an impressive AP of 33.3%, outperforming the widely adopted Mask RCNN with an AP improvement of 8.6%. The success of our proposed method underscores the significance of exploiting the structural properties of pavements and employing efficient data augmentation strategies in crack detection. This research makes a valuable contribution to the field of road maintenance management by providing a more accurate and efficient solution for identifying and segmenting road cracks. Future investigations could focus on further enhancements to our method, including the incorporation of additional contextual information and the exploration of alternative network architectures to further elevate performance levels.

## Figures and Tables

**Table 1:** AP of our proposed method compared to baselines.

Method	Backbone	AP (%)	AP50 (%)	AP75 (%)
Mask R-CNN	R101	24.7	52.2	22.5
Mask Scoring R-CNN		25.2	52.1	22.7
Cascade Mask R-CNN		27.2	52.9	25.5
PointRend		25.3	53.2	23.4
QueryInst		24.2	47.2	24.2
Hybrid Task Cascade		27.8	57.5	27.2
YOLACT		11.6	34.3	5.8
SCNet		26.8	52.4	25.7
Proposed method	ConvNeXt	33.3	59.9	35.8

## Data Availability

The data provided in this work are available from the corresponding author.
